# Effectiveness of Immersive and Embodied Enhancement via “Virtual Reality” on the Sustainability of Hand Hygiene Compliance: Protocol for a Randomized Controlled Trial of a School-Based Training Program

**DOI:** 10.2196/107264

**Published:** 2026-10-06

**Authors:** Siu-lun Chow, King-wa Fu, Isaac Chun-Hai Fung, Patrick Ip, Mike Yat-wah Kwan, Hai Liang, Frank Reichert, Zion Tsz Ho Tse

**Affiliations:** 1 School of Future Media, University of Hong Kong, Room 206, Eliot Hall, University of Hong Kong, Pok Fu Lam, Hong Kong, China (Hong Kong), 852 3917 1643; 2 Department of Biostatistics, Epidemiology, & Environmental Health Sciences, Jiann-Ping Hsu College of Public Health, Georgia Southern University, Statesboro, GA, United States; 3 Department of Paediatrics and Adolescent Medicine, LKS Faculty of Medicine, University of Hong Kong, Hong Kong, China (Hong Kong); 4 Hospital Authority Infectious Disease Centre, Paediatric Infectious Diseases Unit, Princess Margaret Hospital, Hong Kong, China (Hong Kong); 5 School of Journalism and Communication, Chinese University of Hong Kong, Hong Kong, China (Hong Kong); 6 Faculty of Education, University of Hong Kong, Hong Kong, China (Hong Kong); 7 Centre for Bioengineering, Queen Mary University of London, London, England, United Kingdom

**Keywords:** virtual reality, hand hygiene, immersive technology, health promotion, inventions, education, hand disinfection

## Abstract

**Background:**

Regular and sustained handwashing is a key preventive measure for reducing the transmission of infectious diseases among school-aged children. Virtual reality (VR) interventions have demonstrated potential to facilitate behavioral change by creating immersive environments in which behavioral and environmental variables can be systematically manipulated. Compared with conventional verbal or video-based instruction, VR-based handwashing education may provide students with more interactive and experiential learning opportunities, and may improve cleaning of commonly missed areas, such as the wrists. However, evidence on school-based VR interventions for handwashing education in Hong Kong remains limited.

**Objective:**

This study aims to evaluate the effectiveness of a customized VR intervention for teaching handwashing skills to secondary school students in Hong Kong. The intervention teaches the 7-step handwashing technique recommended by the Centre for Health Protection, Department of Health, Hong Kong Special Administrative Region. The study will assess changes in handwashing performance and examine the longer-term effects of the intervention on handwashing habits, behavioral determinants, and hand hygiene.

**Methods:**

A 3-arm randomized controlled trial will be conducted with approximately 500 students allocated to 3 groups on a school-class basis. Each group will receive 2 educational sessions with different interventions over 1 school year (10 months). The first intervention group will receive 2 VR game sessions focusing on handwashing skills. The second intervention group will receive 1 VR game session followed by 1 video session reviewing participants’ gameplay from the first session. The control group will receive 2 video-based handwashing education sessions. Pre- and postintervention UV fluorescence imaging of participants’ hands will serve as the primary outcome measure. The secondary outcomes will be assessed through surveys administered before the intervention, immediately after the intervention, and at a 4-month follow-up. The survey will measure self-efficacy, hand hygiene knowledge, handwashing habits and intentions, attitudes, subjective norms, perceived behavioral control, VR embodiment, and VR experience.

**Results:**

The project was funded in March 2026, and recruitment was conducted from May to August 2026. As of August 2026, 9 schools had confirmed participation, with approximately 500 students expected to be recruited. Data collection is scheduled from September 2026 to June 2027. Data analysis is planned for August 2027, and the study findings are expected to be released in September 2027.

**Conclusions:**

This study will contribute to the development of VR-assisted infection prevention strategies. It addresses several research gaps by developing an evidence-based and feasible VR hand hygiene program for school settings, examining the sustainability of intervention effects, identifying factors and barriers underlying behavioral change in hand hygiene, and exploring handwashing patterns through hand motion data. Findings and implementation experiences from this project may also inform the development of other VR-based health promotion interventions.

## Introduction

### Background

#### Overview

Schools are common epicenters for the transmission of infectious diseases. Outbreaks of COVID-19, influenza, gastrointestinal illnesses, and other communicable diseases have repeatedly shown that students’ everyday hygiene practices can affect not only individual health but also classroom attendance, family well-being, and wider community transmission [1]. Although handwashing is often regarded as a simple personal habit, evidence suggests that appropriate hand hygiene practices among school-aged children and adolescents remain inconsistent. In a multicountry study of adolescents in high school settings, a substantial proportion of students reported inadequate hand hygiene, including washing their hands with soap only sometimes or rarely before eating and after using the toilet [2]. These findings indicate that poor hand hygiene is not merely a problem of knowledge but is also a persistent behavioral issue among young people.

The COVID-19 pandemic further highlighted the importance of hygiene issues in educational settings. Reports by international agencies such as the World Health Organization (WHO) and the United Nations Children's Fund (UNICEF) have emphasized that improving hygiene standards in primary and secondary schools is an urgent component of infectious disease prevention [3]. Cultivating regular and correct handwashing among students is considered a key strategy for reducing the spread of infectious illnesses [4,5]. However, despite the apparent public health significance of hand hygiene, the effectiveness of school-based hand hygiene interventions remains insufficiently established. Earlier systematic reviews have produced mixed findings, while more recent evidence suggests that school programs may reduce infection-related absence, although the behavioral mechanisms behind these effects remain unclear [4]. Researchers have therefore called for larger, longer, and better-controlled cluster trials to determine not only whether the interventions are effective but also how and why they work.

#### Importance of Hand Hygiene in Disease Prevention

Hand hygiene is a foundational infection prevention behavior in both health care and community contexts. The WHO guidelines and implementation tools indicate that improving hand hygiene requires more than health promotion campaigns or unidirectional health communication [6]. A global multisite study of the WHO multimodal strategy demonstrated that hand hygiene compliance can be improved when interventions are structured, sustained, and supported by multiple components [7]. This suggests that hand hygiene behavior is modifiable, but it requires a carefully designed intervention rather than information provision alone.

Good hand hygiene involves several interrelated components. Individuals must recognize when handwashing is required, such as before eating, after using the toilet, after coughing or sneezing, or after contacting potentially contaminated surfaces. In the health care context, this is often promoted via the WHO “My Five Moments for Hand Hygiene”; in community and school settings, a similar educational campaign is required. Furthermore, quality standards of handwashing are also essential. To maximize disease prevention, individuals should perform handwashing correctly, covering key hand surfaces and following the official procedural steps [8]. Adequate time, sufficient duration, and thorough coverage of handwashing are also critical. Finally, knowledge, motivation, and behavioral control are key factors in sustaining these practices over time. These multiple requirements explain why hand hygiene should be understood as a complex behavioral practice rather than a simple act of washing hands.

#### Virtual Reality Virtual RealityExperience and Behavioral Training

The increasing affordability and usability of virtual reality Virtual Reality (VR) devices make this research especially timely. Modern headsets are less expensive, more portable, and easier to operate than earlier generations of VR equipment. In health care training, VR has been used to provide safe rehearsal of rare, risky, or complex scenarios, including infection exposure. Recent studies have evaluated VR hand hygiene training among health care assistants, nurses, infection-control personnel, and food handlers [9-11]. These studies commonly report improvements in execution, timing, self-efficacy, adherence, or user satisfaction. For instance, a VR program based on the WHO “My Five Moments” framework simulated clinical scenarios and visualized pathogen transmission, helping nurses improve self-efficacy and hand hygiene adherence [9]. Another study among food handlers found VR training to be more effective than conventional face-to-face instruction [10]. Broader reviews of VR-based health care education also suggest gains in knowledge, skills, and attitudes, although the extent of outcomes depends on implementation contexts [12].

The interventional effect of VR can be explained through learning science and behavior change mechanisms. One common reason for hand hygiene failure is the absence of immediate feedback. In daily life, germs are invisible, and the consequences of poor handwashing are delayed or uncertain. VR can overcome this by making contamination visible and by showing users immediately when they make mistakes. For example, simulated germs remain on unwashed areas of the hands, or pathogen transmission is shown after touching a contaminated surface. This strengthens associative learning by linking action, error, and consequence within the same experience. It may also increase intrinsic motivation because users do not passively receive information but actively adjust their own behaviors [13].

VR also has advantages in standardization and scalability. In conventional instructor-led training, demonstrations, explanations, and feedback may vary across teachers, schools, and sessions. When an intervention is delivered across multiple sites, such variation can reduce fidelity. A VR program, in contrast, delivers the same content, scoring system, and feedback structure across all participants. This is particularly valuable for behavioral training that requires consistency in instruction and assessment. VR systems can also collect detailed performance data, including duration, sequence, movement, missed areas, errors, and repeated attempts. With appropriate validation, such data can provide more objective measures of procedural performance than self-reported behaviors [14,15].

Recent devices further increase the potential of VR-based hand hygiene research. The latest headsets include hand tracking technology capable of capturing detailed hand movement, including finger positions, rotations, and spatial movement patterns [16,17]. Although the validity of VR-derived measures depends on device accuracy and scoring algorithms, new systems have shown promising robustness and precision. For a handwashing intervention, this opens the possibility of measuring not only the self-reported handwashing pattern but also the actual hand movement during the learning task.

#### Presence and Embodiment

Two immersive VR features are especially relevant to behavior change: presence and embodiment. Presence refers to the subjective psychological sense of “being there” inside a virtual environment, describing the user’s psychological response to the technical features of the VR system, such as visual fidelity, interactivity, and sensory richness. When presence is strong, users may respond to virtual events as if they are meaningful and personally relevant. In hand hygiene training, this means that simulated contamination, infection risk, or incomplete washing may feel more immediate than the same information presented in a lecture [18-20].

Embodiment is another important dimension of immersive learning. It refers to the sense of having or inhabiting a body part in the VR system. For example, realistic hand representation can create a sense of ownership over virtual hands, particularly when users see their own movements reflected in real time. First-person perspective and responsive hand tracking can strengthen this effect. In handwashing education, embodiment is particularly important because the target behavior is bodily and procedural. Students are not simply learning a concept; they are learning how to move, rub, cover, and sequence their hands. A virtual representation of their hands therefore helps them connect abstract hygiene knowledge with bodily practice [21-23].

The combination of presence and embodiment makes VR especially suitable for handwashing education. Traditional noninteractive interventions often rely on posters, lectures, videos, or demonstrations. These can convey information, but they may not fully engage students in the experience of contamination and correction. In contrast, immersive and embodied VR shows students how germs spread, where washing is incomplete, and how improved technique changes the outcome.

#### Theory of Planned Behavior

The theory of planned behavior (TPB) provides a useful framework for examining how VR influences hand hygiene. It posits that intention and behavior are shaped by attitude, subjective norms, and perceived behavioral control. In handwashing, attitude refers to the extent to which the participants believe hand hygiene is useful, important, or worthwhile [24,25]. Subjective norm represents the extent to which they believe significant others, such as parents, teachers, classmates, or society, expect them to wash their hands properly. Perceived behavioral control denotes the degree to which they feel capable of performing the behavior under real conditions. VR may influence all 3 domains: it can make the benefits of handwashing more vivid, show the social consequences of contamination, and increase confidence through practice.

TPB also supports local contextualization. A Hong Kong study found that subjective norm and perceived behavioral control were important predictors of nurses’ hand hygiene behaviors [26]. This may reflect cultural and social patterns where behavior can be strongly influenced by the expectations of significant others. In school settings, this may be especially relevant because of the influences of peer behavior, teacher expectations, school routines, and family norms. A VR intervention designed for Hong Kong students should therefore examine not only individual knowledge but also perceived norms, confidence, and intention [27].

#### Effectiveness of VR Hand Hygiene Interventions

Nevertheless, relatively little is known about the effectiveness of VR hand hygiene interventions in community and school settings. Existing studies are often small, nonrandomized, or focused on health care workers. For example, Shimada et al [28] reported that a VR handwashing education system may help preschool children wash more thoroughly than conventional verbal explanation, particularly in difficult-to-wash areas such as the wrists. Although these findings are encouraging, the evidence remains preliminary. A well-designed, school-based intervention with adequate sample size, randomization, and follow-up is still lacking.

Another limitation of existing hand hygiene interventions is on the sustainability of handwashing behavior after the interventions. Many programs demonstrate immediate or short-term enhancement, but it remains uncertain whether behavioral change persists beyond the initial intervention period. This is a particularly important issue because infectious disease prevention depends on repeated practice, not one-time learning. Like many clinical and educational trials with short follow-up periods, existing hand hygiene studies often focus on immediate knowledge gain or short-term technique improvement. Systematic reviews rarely address whether the behavior is maintained over time. This creates a significant research gap.

VR may offer one possible solution to this sustainability problem. As VR is immersive and interactive, it may produce stronger learning experiences than lecture-based or image-based instruction. Birrenbach et al [29], for example, found that VR simulation was comparable to traditional video and written instruction for teaching hand hygiene skills to medical students, while also producing higher user satisfaction and a more sustained training effect. These findings suggest that VR may not simply teach procedures but may also create memorable experiences that support longer-term behavioral reinforcement. Further research is therefore needed to test whether VR can generate sustained improvement in hand hygiene among school students.

In the context of Hong Kong, VR has only recently begun to enter educational and medical training environments. Local implementation should therefore consider not only effectiveness but also acceptability, usability, implementation logistics, and the costs and benefits of the intervention. Previous studies evaluating VR hand hygiene training in health care settings have shown that user acceptance is critical: even an effective intervention may fail if participants find the headset uncomfortable, the environment unrealistic, or the logistics too demanding [30]. Similar research has also suggested that the design of the virtual environment, including its realism and relevance to the user’s context, may influence training outcomes [31]. Therefore, a school-based VR intervention should examine both behavioral effects and implementation feasibility.

Overall, this study addresses an important gap in hand hygiene intervention research. Handwashing is a critical public health behavior, but existing school-based interventions provide limited evidence on long-term sustainability and behavioral mechanisms. VR offers a theoretically grounded and technologically feasible intervention modality because it can visualize invisible contamination, support embodied practice, provide immediate feedback, standardize delivery, and generate objective performance data. At the same time, its effectiveness, acceptability, and sustainability in Hong Kong school settings remain underexplored. By integrating VR learning with behavioral theory and local implementation considerations, this study aims to evaluate whether immersive hand hygiene training can improve students’ knowledge, self-efficacy, intention, and observed handwashing performance and whether these effects sustain over time.

### Objectives

This study aims to evaluate the effectiveness of a VR intervention for teaching handwashing skills. A 7-step handwashing approach, which is promoted by the Centre for Health Protection, Department of Health, Hong Kong Special Administrative Region (HKSAR), will be taught through a customized VR game in secondary schools in Hong Kong. Participants’ handwashing skills and the prolonged effects on handwashing habits, knowledge, attitude, and intention will be assessed. This study also investigates the determinants of behavior change related to the pathway of and barriers to learning handwashing through VR devices.

### Hypothesis

This study hypothesizes that participants in the VR intervention groups will have better handwashing skills than those in the control group after the intervention. This will be evident from hand images obtained using a UV light test after handwashing at the pre- and post-stages of the whole research study. The participants in the 2 intervention groups are also hypothesized to have better handwashing knowledge, habits, attitudes, and intentions than the control group. The relevant measurements will be collected through questionnaires at different time points during the study. Meanwhile, we also hypothesize that the first intervention group, which receives more VR sessions, will outperform the second intervention group, which receives fewer VR sessions (ie, one of the VR sessions is substituted by a recorded video session of handwashing).

We hypothesize that measurements of TPB, immersive tendency, sense of presence, and sense of VR embodiment will be mediators of participants’ handwashing improvement.

## Methods

### Study Design

A 3-arm randomized controlled trial will be conducted in accordance with the SPIRIT (Standard Protocol Items: Recommendations for Interventional Trials) checklist (Checklist 1). Approximately 500 students will be assigned to 3 groups, which will be randomly assigned on a class basis. Each group will receive 2 sessions of educational training with different kinds of interventions throughout 1 school year (10 months). Intervention group 1 (full intervention) will participate in 2 VR game sessions on handwashing skills; intervention group 2 (half intervention) will participate in 1 VR game session and 1 video session reviewing their handwashing playthrough captured in the first VR game session; and the baseline (control) group will participate in 2 video sessions, which broadcast official promotional videos of handwashing steps. The 2 training sessions will be 6 months apart.

Assessments will be made at 4 points: preintervention (T0), immediately after the first training session (T1), immediately after the second training session (T2), and at the final follow-up assessment 4 months after the second training session (T3; Table 1). Owing to administrative burden and budget limitations, the hand imaging using a UV light test will only be conducted at T0, T1, and T3. All the questionnaires will be administered at T0, T1, T2, and T3, but questions related to sense of presence and embodiment will be only inquired with the intervention groups because the control group does not participate in the handwashing VR game. Table 1 summarizes the assessments.

**Table 1.** Summary of assessments.

Assessment	Preintervention (September 2026)		Post-first training session (September 2026)		Post-second training session (March 2027)		Follow-up (July 2027)	
	Intervention	Control	Intervention	Control	Intervention	Control	Intervention	Control
Hand images	✓	✓	✓	✓			✓	✓
Habit	✓	✓	✓	✓	✓	✓	✓	✓
Attitude and intention	✓	✓	✓	✓	✓	✓	✓	✓
TPB^a^	✓	✓	✓	✓	✓	✓	✓	✓
Immersive tendency	✓	✓	✓	✓	✓	✓	✓	✓
Presences	✓		✓		✓		✓	
Embodiment	✓		✓		✓		✓	

a TPB: theory of planned behavior.

All the trial activities will be conducted in the classrooms of secondary schools.

### Ethical Considerations

The protocol for this study has been registered with ClinicalTrials.gov (identifier: NCT07659340). This research project has also obtained ethical approval from the Human Research Ethics Committee, the University of Hong Kong (HKU; reference EA250574). The trial and data collection are being monitored by the Human Research Ethics Committee.

Schools will only provide student numbers and class numbers to the research team for assigning trial groups. The research team will then assign a nonidentifiable pseudo-ID to the students. School administrators will inform the students of their pseudo-IDs as identifiers throughout this research project.

Withdrawal from the study is voluntary at any time without any induced consequences.

### Participant Recruitment

This study targets secondary school students in junior forms (S1 to S4) in Hong Kong, who are typically aged 12 to 15 years. Potential participants will be recruited from secondary schools in Hong Kong.

Invitations to join this research study have been sent to all secondary schools in Hong Kong via email in December 2025, March 2026, and June 2026.

The research team will screen enrolled schools to ensure that (1) schools that participated in this study cover the 3 major geographical areas (Hong Kong Island, Kowloon, and the New Territories) of Hong Kong; (2) classes are selected from different junior forms to maintain age variability; and (3) schools come from different academic performance “bands” (assigned by the Education Bureau based on schools’ academic performance) to capture variability in students’ learning ability in the samples.

Recruitment commenced in March 2026 and will continue until September 2026. Schools enrolled will contribute 2 to 3 classes (normally 20-30 students per class in Hong Kong) to this study. The trial will be conducted from September 2026 to June 2027.

After the completion of the whole study, participating schools will be awarded a certificate of commendation and a HK $2000 (US $254.94) book voucher. Every student who completes the whole study will also be awarded a HK $100 (US $12.75) gift voucher.

### Sample Size Estimation

Cohen power analysis for multiple regression is used to estimate the required sample size. We assume a statistical power of 0.80, a significance level of 0.05, a medium effect size (Cohen *f*^2^=0.15) as our prerequisite, and approximately 10 predictors in our final model (including gender, socioeconomic factors, treatment effect, and phase effects for each phase). Under these assumptions, the smallest total sample size that achieves power ≥0.80 is about 118 (calculated using the R package pwr).

Nevertheless, due to administrative feasibility, we may not be able to randomize individual students into the 3 groups designed in the proposal. The assignment will be class based, and such class-based clustering in randomization leads to statistical power loss because within-class participants are expected to be more correlated than participants across classes. Therefore, a compensatory approach is introduced to consider such design effect by increasing the sample size, which is quantified by the ratio of the number of participants required in the cluster trial to the number of participants needed using individual randomization.

The formula for the ratio is as follows [32]:


DEFF=1+(m−1)×ICC

where DEFF is the design effect, m is the average cluster size (number of participants per cluster), and ICC is the intraclass correlation coefficient.

We assume 25 students (per class) offered by participating schools, and the ICC is conservatively assumed to be 0.10, which is drawn from the literature on ICC between educational institutes [33]. The design effect is computed as 3.9; therefore, the basic sample size required is at least 118×3.9≈400. As we also assume approximately 20% nonresponse across all assessments or dropout during the intervention, we will recruit approximately 500 students for the whole study.

### Registration and Research Consent

Before the intervention, printed consent forms will be distributed to parents to obtain their consent for their children to (1) participate in the game sessions (intervention) and (2) participate in the fluorescent lotion and UV lighting test. Permissions for collecting hand images, hand motions, and questionnaire data will also be sought from the parents of participating students. Participation in the assessments will not affect the VR activities delivered by the research team. Assent forms will also be distributed to participating students. This study does not involve drug use or medical treatment, and there are no anticipated physical or medical risks to participants.

Some participants may encounter sickness and discomfort (eg, headache) after using the head-mounted VR devices, but such reactions are rare. Participants can cease playing the game at any time during the intervention activities.

### Randomization

Randomization of classes will be conducted using well-developed Python-based software (eg, MinimPy). The randomization will be implemented by a researcher with a statistical background training. Each class will be randomly given a pseudo-number and randomly assigned to one of the three groups: (1) the full intervention group, (2) the half intervention group, or (3) the control group. No individual information about students will be collected before the preintervention assessment.

Only the project manager and the researcher with statistical background training will know the randomization sequence. To maintain concealment, the randomization sequence will only be disclosed to schools for administration purposes until the intervention commences. After assignment, enrolled participants will be blinded to the assigned group until the first training (intervention) session.

### Intervention

The VR game will teach the “7-step approach” to handwashing, which is based on the health promotion campaign of the Centre for Health Protection, HKSAR. The game comprises 2 parts: training mode and challenge mode. In training mode, students will be guided by a pair of on-screen “ghost hands,” textual instruction, and with the support of voice guidance. The pair of “ghost hands” will demonstrate how to perform the 7 steps of handwashing. Students will follow the motions of the “ghost hands” to complete the handwashing tasks. In each handwashing step, a determinate progress bar will be displayed; it will be filled from 0% to 100% when the participants’ hand motions are continuously similar to the “ghost hand.” In the challenge mode, students will be requested to repeat all the steps at once from memory, and hints will be given only when they encounter a setback within an assigned duration.

Trained facilitators will teach participants how to use the VR devices and provide briefing sessions on how to play the game in advance.

Intervention group 1 will be trained through 2 VR game sessions, and intervention group 2 will be trained through 1 VR game session and then 1 video session on videos captured from the playthrough of the first game session. This setting can test whether successive VR training will be more effective than a single VR game session mixed with a recorded video of the previous VR experience.

The control group will not participate in any VR handwashing game during the study period but instead will receive 2 video watching sessions regarding handwashing. The videos used were originally broadcast by the Centre for Health Protection (eg, [34]). The videos will be played in the 2 sessions to enable the participants in the control group to receive the official health promotion campaign messages of hand hygiene and the handwashing steps. To compensate participants in the control group, the research team will provide them with extra VR game sessions after the study period (Figure 1).

**Figure 1.**
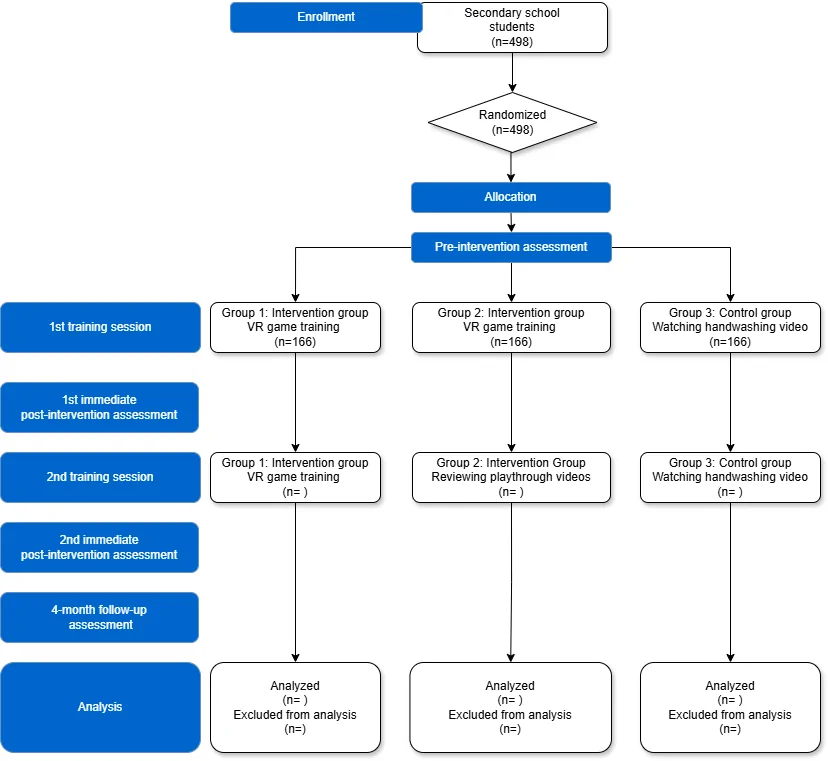
Randomized controlled trial CONSORT (Consolidated Standards of Reporting Trials) flow diagram for enrollment, intervention, assessment, and analysis. VR: virtual reality.

### Outcome Measures

#### Primary Measure: Hand Images

Participants’ handwashing performance will be assessed by statistical analysis of hand images. UV-sensitive fluorescent lotion (eg, Glo Germ) will be applied thoroughly to the participants’ hands to simulate full contamination (eg, pathogens) on the hands. Participants will then wash their hands. The areas where the lotion has been washed out are considered “decontaminated.” The participants will put their washed hands in a black box with an UV-A (365‐395 nm) lighting system. Both palms and dorsa of the left and right hands will be photographed. This measure will be implemented at the beginning and end of the intervention. This method of assessment has been used in previous studies [35-38]. Besides making pre- and post-comparison, this method also indicates which parts of the hands are more likely to be missed in the handwashing processes [39,40].

#### Secondary Measures

##### Knowledge of Hand Hygiene and Handwashing

This study focuses on handwashing skills and steps; questionnaires in this field are usually customized for the specific research context (ie, steps and skills can be different between studies). For example, Gasteiger et al’s [31] study asks how long hand hygiene should be practiced (in terms of washing duration). As our interventions mainly concern the handwashing steps, 4 questions are developed to assess participants’ specific knowledge of the handwashing procedures. The sample question asks which is the correct handwashing step, with a correct answer and other distractor choices. We will also ask what should be avoided during handwashing.

##### Handwashing Habit

Handwashing habits will be measured as the self-reported frequency of handwashing. Five items on how often the participants cleaned their hands in 5 different scenarios (before meals, after using the washroom, after taking public transportation, after arriving at school, and after going back home) will be used to measure the frequency of handwashing (5-point scale from “never” to “always”).

##### Attitude and Intention

The attitude toward automaticity of handwashing habits will be measured by the Self-Report Behavior Automaticity Index [41,42], which has already been modified and validated into 4 items related to handwashing and hand hygiene in the study by Diefenbacher et al [43]. A sample item in Diefenbacher et al’s study is “Hand washing is something I start doing before I realize I‘m doing it.” The 4 items are measured on a 7-point Likert scale, ranging from 1 (“does not apply to me at all”) to 7 (“completely applies to me”). Behavioral intention will be measured by 2 items extracted from Diefenbacher et al [43] and Baretta et al [44] (eg, “In my daily routine, I want to perform hand hygiene as correctly as possible”) on a 7-point Likert scale, and another 2 items designed by our team to gauge whether intention will be undermined under certain conditions (eg, “I intend to wash my hands after using the washroom even though my hands feel clean.”).

### Mediators and Evaluative Items

#### Scale for TPB

Three central dimensions of TPB will be gauged, namely, subjective norm, perceived behavioral control, and barriers [45], using a Likert scale, ranging from 1 (strongly disagree) to 5 (strongly agree). Three items correspond to subjective norms (eg, “Handwashing is an effective way to protect me from getting infected by diseases”). Three items correspond to perceived behavioral control (eg, “People around me will have a lower risk of contamination if I have a good hand hygiene habit”), and 5 items correspond to barriers (eg, “I am not clear about the whole protocol of hand washing or hand rubbing”). All items are extracted and modified from studies by White et al [46], Jeong and Kim [47], and Wollast et al [48], which adopted scales from TPB to investigate hand hygiene practices. Applicable items are modified into the context of student and school settings.

#### Immersive Tendency

Immersive tendency has been measured and validated by Jerome and Witmer [49]. The 9-item scale is rated on a 5-point Likert scale; 4 items range from 1 (strongly disagree) to 5 (strongly agree), and another 5 items range from 1 (never) to 5 (always).

#### Presence

Measurement of presence is also extracted from Jerome and Witmer’s study, consisting of 13 items [49]. All items are rated on a 5-point Likert scale, ranging from 1 (strongly disagree) to 5 (strongly agree). This measurement will be implemented only with the 2 intervention groups because the scale probes the VR gaming experience.

#### VR Embodiment

To assess participants’ senses of embodiment in their VR experience, 6 items are adopted from the study by Galvan Debarba et al [23]. The items probe participants’ senses of agency, body ownership, and the sense of engaging in the game environment. Of the original items, 2 were related to “floor fall feeling” since the game in the study by Galvan Debarba et al [23] offered participants a VR floor collapse experience, and another 2 were about the sense of “location.” Our study will remove these 4 items and modify the remaining items into asking handwashing VR experience. All items are rated on a 7-point Likert scale, ranging from 1 (“strongly disagree”) to 7 (“strongly agree”). Our study adds 1 more question about the feeling of virtual germs on the hands. This part of the scale will be implemented only with the 2 intervention groups because the scale probes the VR game experience.

### Data Collection

#### Hand Imaging Data

Hand imaging data obtained from the main measurement will only be collected before and after the first intervention (T0 and T1) and at the end of the data collection period (T3, about 10 months after the first intervention).

Hand imaging data will be saved as JPEGs and converted into a pixel matrix.

#### Quantitative Survey Data

Self-administered surveys will be deployed at the beginning of the first training session, immediately after the first training session, immediately after the second training session, and 4 months after the second training session. The survey comprises scales measuring knowledge of hand hygiene, handwashing habits, attitude and intention, subjective norms, and perceived behavioral control, VR embodiment, and other VR experience. The survey will be distributed through the Qualtrics platform to participants’ school email accounts. Each participant will receive a unique survey link, and they can complete the survey online. School teachers will help supervise students to complete the survey on time.

Survey data will be further cleaned and checked for validity by researchers of the project. Missing data will be handled using full information maximum likelihood estimation.

A separate network-attached storage device with a security password has been established at HKU for secure data storage of this research project. Only the principal investigator and the project manager will know the IP address and the password of the network-attached storage device.

### Statistical Analysis

Each participant’s pre- and post-study hand images will be compared based on the residual fluorescence percentage, which is calculated as fluorescent pixels over total hand pixels. These percentages serve as a handwashing index, and a lower fluorescent percentage indicates better performance (better decontamination).

Linear mixed effects models will be used to evaluate whether the index changes significantly between treatment groups and the control group after the intervention.

The model uses an unstructured covariance matrix, which means that variance may be different at each time point and correlations between measurement times may be different for each time pairing. This assumption is made because the period between different assessments is of different durations. The model can be formulated as follows:


$$Y_{tij}=\beta_{0}+\beta_{1}({TIME}_{tij})+\beta_{2}({GROUP}_{j})+\beta_{3}({TIME}_{tij}\times {GROUP}_{j})+u_{0j}+b_{0ij}+b_{1ij}({TIME}_{tij})+\varepsilon_{tij}$$

In this formula, Y_*tij*_ is the outcome of student *i* at time *t* from class *j*, β_0_ is the population estimate of the intercept for the control group, β_1_ is the population estimate of the linear slope for the control group (ie, GROUP_*j*_=0, when class *j* is in the control group), β_2_ and β_3_ are the estimates of the mean difference in intercept and slope, respectively, between conditions (ie, control group: GROUP_*j*_=0; half treatment: GROUP_*j*_=1; and full treatment: GROUP_*j*_=2), *u*_0j_ is the class-specific random slope, *b*_0*ij*_ is the initial level of student *i* (individual-specific intercept), *b*_1*i*_ is the growth rate of student *i* (individual-specific slope), and ε_*tij*_ is the time-specific residual. This model is sometimes regarded as a conditional growth model in which random intercepts and slopes are conditioned on predictor variables [50]. The goal of this study is to test whether β_3_ (ie, the group×time interaction) is significantly different from 0. The primary measure and the individual secondary measure will be treated as dependent variables in separate models. As the number of clusters in this study may be small (eg, 15-20 school classes), restricted maximum likelihood procedures will be applied in fitting the model, and the Kenward–Roger approximation will be adopted to adjust the degrees of freedom, rather than relying on a traditional asymptotic z-test [51]. The calculation will be performed using the R package lme4, with additional packages such as *hlmLab* for data management and visualization.

## Results

### Overview

The project has been funded since March 2026. Participant recruitment was conducted from May to August 2026. As of August 2026, a total of 9 schools had agreed to join the project, and approximately 550 students are expected to be recruited from these schools. Data collection will start in mid-September 2026 and will be completed in June 2027. Data will be cleaned and analyzed in July and August 2027. Preliminary results are expected to be released in September 2027.

### Dissemination Plans

A final report of the whole research project will be submitted to the funder in September 2027 and will be made publicly available on the funder’s website. Complete research results will be published in the form of journal papers.

### Data Sharing

Schools will only provide student numbers and class numbers to the research team for assigning trial groups, and then the research team will assign a nonidentifiable pseudo-ID to the students. School administrators will inform the students of their pseudo-IDs used in this research project. The research team will not know the names and personal particulars of individual students, nor will they impute any identifiable personal particulars in the dataset.

Anonymized data will be shared on the HKU Data Hub [52]. This is a data repository backed up by Figshare, where research data produced by HKU researchers and collaborators are hosted. Individuals and participating schools will be anonymous. Only a data matrix with pseudo-IDs of the participants and the schools will be uploaded to the repository.

## Discussion

### Anticipated Findings

This study aims to evaluate the effectiveness of a VR game intervention in enhancing secondary students’ handwashing skills. The results of this study will contribute to high-quality delivery of the VR game intervention by tracking participants’ handwashing skills, habits, attitudes, and intentions. We expect that intervention group 1 will have better enhancement in handwashing skills, measured by captured hand images, than intervention group 2 and the control group because participants in intervention group 1 will receive a higher dosage of VR game sessions than participants in the other 2 groups. Similarly, for the secondary measures (knowledge, habits, attitudes, and intentions), intervention group 1 is expected to demonstrate better results than intervention group 2 and the control group. Meanwhile, the 2 intervention groups will perform better than the control group on all the measurements at the end of the study.

This study will be run for 10 months with successive training and assessment at different time points. We hope to gauge the sustainability of the intervention impact over a longer period on handwashing behaviors. We expect the intervention will have a more prolonged effect in intervention group 1 than in intervention group 2.

By imposing a set of mediators, such as items extracted from the TPB, immersive tendency, sense of presence, and sense of embodiment during the VR experience, we expect to identify the determinants of behavior change related to the mechanisms of and barriers to handwashing practice. The preliminary anticipation is that the more VR sessions the participants participate in, the more aware they will be of subjective norms and perceived behavioral control and the fewer barriers to performing hand hygiene, from the perspective of TPB. The immersive tendency, sense of presence, and sense of embodiment will also be enhanced when the participants engage in more VR sessions.

### Strengths and Limitations

This study can reveal whether VR games can foster students’ satisfaction with the learning process and then maintain a more sustained effect after the training.

To strike a balance between the treatment effect and the cost of service delivery, the UV fluorescence hand images will only be measured at T0, T1, and T3. Participants’ changes in handwashing skills between these time points can be reflected only by secondary measures.

Another limitation is that not every student has prior VR gaming experience. Although our research team will train some facilitators to assist participants in using the VR device and briefing sessions will also be delivered, participants who may feel very unfamiliar or uncomfortable using VR headsets may drop out of this study. Nevertheless, this is also part of the results, as this research project also examines the acceptance of VR experience in the learning process. The extent of this kind of setback will be reported in the final report.

#### Conclusions

This study will contribute to the development of infection prevention strategies through a VR game. Compared with other VR hand hygiene studies [11,13,22,27], this study fills a number of research gaps in the field by (1) developing evidence-based and executable VR hand hygiene programs in school settings; (2) addressing the sustainability issue of hand hygiene intervention effects; (3) identifying factors underlying the pathway of behavioral changes in hand hygiene and issues related to barriers to change; and (4) identifying handwashing patterns through hand motion data shaping the hand hygiene level. The experiences and results from this project will be transferable to the development of other health promotion programs by adopting VR devices.

## Supplementary material

10.2196/107264 Checklist 1 SPIRIT checklist.

10.2196/107264 Peer Review Report 1 Funding and protocol assessment report.

## Abbreviations

HKSAR: Hong Kong Special Administrative Region
HKU: University of Hong Kong
ICC: intraclass correlation coefficient
SPIRIT: Standard Protocol Items: Recommendations for Interventional Trials
TPB: theory of planned behavior
UNICEF: United Nations Children's Fund
VR: virtual reality
WHO: World Health Organization
