# Supplementary material for: Effectiveness of Immersive and Embodied Enhancement via “Virtual Reality” on the Sustainability of Hand Hygiene Compliance: Protocol for a Randomized Controlled Trial of a School-Based Training Program
Source: JMIR Res Protoc. 2026 Oct 6;15:e107264. doi: 10.2196/107264 (PMC13642894; doi:10.2196/107264)
Supplement: Supplementary file 1 [file resprot-v15-e107264-s001.pdf]

Administrative information	
**Title**	P.1
**Structured summary:**	P.1 – P.2
**Protocol version**	P.2
**Roles and responsibilities**	**Trial Sponsor:**<br>P.24
**Open Science**	
**Trial registration**	**NCT07659340**
**Protocol and statistical analysis plan**	P.18-20
**Data sharing**	P.20
**Funding and conflicts of interest**	P.23
**Dissemination policy**	P.20
**Introduction**	
**Background and rationale**	P.3-P.9
**Objectives**	P.9
**Methods: Patient and public involvement, trial design**	
**Patient and public involvement**	Nil.
**Trial design**	P.10-P.11

**Trial setting**	P.11
**Eligibility criteria**	P.11
**Intervention and comparator**	P.14
**Outcomes**	P.15-18
**Harms**	P.13
**Participant timeline**	P.15
**Sample size**	P.12-P.13
**Recruitment**	P.12
**Methods: Assignment of interventions**	
**Sequence generation**	P.13
**Allocation concealment mechanism**	P.13
**Implementation**	P.13
**Blinding**	P.13
**Methods: Data collection, management, and analysis**	
**Data collection methods**	P.18-19
**Data management**	P.19
**Statistical methods**	P.19-P.20
**Methods: Monitoring**	

**Data monitoring committee**	P.11
**Trial monitoring**	
**Methods: Monitoring**	
**Research ethics approval**	P.11
**Protocol amendments**	Nil
**Consent or assent**	P.13
**Confidentiality**	P.11
**Ancillary and post-trial care**	No ancillary or post-trial care is needed in this study
