# Supplementary material for: Effectiveness of Immersive and Embodied Enhancement via “Virtual Reality” on the Sustainability of Hand Hygiene Compliance: Protocol for a Randomized Controlled Trial of a School-Based Training Program
Source: JMIR Res Protoc. 2026 Oct 6;15:e107264. doi: 10.2196/107264 (PMC13642894; doi:10.2196/107264)
Supplement: Supplementary file 2 [file resprot-v15-e107264-s002.pdf]

Research Council

Grant Review Board (GRB)

GRB ASSESSMENT REPORT – GRANT APPLICATION

Application No. : 23244181

Project Title : Effectiveness of Immersive and Embodied Enhancement via "Virtual Reality" on the Sustainability of Hand Hygiene Compliance: School-based RCT with 10-month Follow-up

Principal Applicant (PA) : Prof FU KING WA

Rating : 4. Recommended for support

GRB's comments

1. The resubmission has addressed most of the previous comments.

2. Justify the sample size calculation and clarify the primary and secondary outcomes (see Reviewer 4's comments for details).

3. Consider to include an additional 3-month "booster" reminders (see Reviewer 3's comments for details).

4. Provide evidence such as a letter of support from all participating schools to support the feasibility of subject recruitment.

5. Declare whether there is any similar/related research work done, on-going and to be done (since submission of this application) in relation to the proposal before the proposed start date stated in the application form (Section 11). Such declaration does not necessarily mean that the proposal concerned will be adversely affected. However, any such non-disclosure may be subject to penalty. Provide details of the work done and submit a revised proposal (Section 10) with changes in related budget under Section 10f (Justification of Requirements), if any. Changes in the grant proposal will be sent to GRB for review and endorsement, if applicable.

6. Confirm whether the proposed "Project Coordinator" is either "Senior Research Assistant" or "Post-doctoral Fellow", and revise both the budget and Section 10f (Justification of Requirements) accordingly to reflect the confirmed rank. If the "Project Coordinator" is a Post-doctoral Fellow, confirm that this post allocated with 100% project effort, is not supported by other funding sources (e.g. University Grants Committee/Research Grants Council). Additionally, ensure that the post title is consistently used across both Section 14 and Section 10f to avoid confusion.

7. Clarify if the proposed helpers are student helpers. If yes, confirm whether the proposed student helpers will enter into employment contracts with the Administering Institution (AI) or engage in AI's engagement scheme for student helpers/casual helpers which is bound by the Employment Ordinance. If not, categorise it under "Other Expenses".

8. Provide justification and quotation(s) as supporting document(s) for the following budget items: "VR headset and finger tracker" ($105,000). Confirm whether the budget item adheres to the established procurement policy of the Administering Institution.

9. Provide a detailed justification for the following budget item: "Certificate participation printing" ($8,000). Incentives/tokens of appreciation for study participants are usually gifts or coupons ranging from $20 to $200, depending on the level of participation.

10. Human research ethics approval from a recognised ethics committee is required.

Research Council

Grant Review Board (GRB)

Reviewers' comments

- Respond to the Reviewers' comments in the following pages point-by-point.

Action(s) required by PA

- Refer to the checklist at https://rfs.healthbureau.gov.hk/images/HMRF/Checklist_for_fundable_projects.pdf for details in preparing your response.
- Respond to both GRB's and Reviewers' comments point-by-point.
- Submit a revised "Research Project Proposal", i.e. Section 10 of Application Form, in pdf files (both "track change" version and clean copy are required).
- Submit a revised budget plan under Sections 14a to 14d of the application form.
- Submit all the materials required via eGMS by 15 October 2025.
- Submit (a) safety/ethics approval(s)/evidence for accessing third-party data (all documents need to bear same project title as in the application)/regulatory approval, if applicable; (b) personal email address; and (c) mobile number via eGMS no later than 17 December 2025.

Grading for applications	Meaning
**4**<br>**Recommended for support**	Nil or very minor issues to address only
**3**<br>**Recommended for support**<br>**subject to clarifications / amendments**	Minor revision and clarification required for a successful delivery
**2**<br>**Not recommended for support**<br>**at present**	Major revision required for significant improvement
**1**<br>**Not supported**	Minimal impact on research/flaw in methodology/incomplete application/ out of scope of the fund

REFEREE'S ASSESSMENT FORM

Reference No.: 23244181

Project Title: Effectiveness of Immersive and Embodied Enhancement via "Virtual Reality" on the Sustainability of Hand Hygiene Compliance: School-based RCT with 10-month Follow-up

PART A: REFEREE'S DETAILED REMARKS ON THE INDIVIDUAL SECTIONS OF THE GRANT APPLICATION

1. **Originality and Impact** What is the importance of the proposed research in terms of its originality and potential impact in the area under study? How will the research findings benefit patients and/or the healthcare system? Will the research findings improve patient care, population health, influence clinical practice and/or health services management, or inform health policy in Hong Kong and elsewhere? Have the potential facilitators and barriers to this impact being achieved been identified?

As I stated previously, this is an original and very interesting study, given the gap in the effectiveness of this type of technology in achieving behavioral change.

2. **Research Questions, Aims and Hypotheses** How specific, clearly expressed and realistic are the research questions, aims and hypotheses?

The research questions, aims and hypotheses are well explained, realistic and proper. By simplifying the intervention arms, the study has further improved its quality in my opinion.

3. **Subjects and Study Methodology** (i) Is the proposed design and methodology appropriate for the study? (ii) Are sample sizes clear, justified, adequate and realistic? (iii) Are any preliminary data available? (iv) How feasible is the proposed timeframe? (v) Please also provide comments on the following (where applicable):

- For proposals submitted under Advanced Medical Research (refer to Area of Project on page 1 of application), is this a clinical study which applies advanced technologies including but not limited to biotechnology in medicine, use of drugs and treatments, clinical trials, virtual health such as telemedicine, etc., to facilitate the translation of knowledge generated from health and health services or infectious diseases studies into clinical practice and to inform health policy?
- For proposals addressing thematic priorities under Implementation Science (refer to Section 5b of the application), are the appropriate framework(s) / model(s) with the pre-set criteria proposed to evaluate/assess the barriers and facilitators of implementation outcomes clearly stated?
- For Seed Grant proposals (i.e. grant ceiling is HK$500,000), is the prospect that a successful outcome will enable scale-up to a larger project/trial and/or enhance the efficacy/effectiveness of existing practice clearly stated and feasible?

The methodology is appropriate, and the problems encountered in the previous version have been resolved, both in terms of sample size calculation and the randomization of intervention groups.

4. **Outcomes and Data Analysis** (i) Are the primary and secondary outcomes clearly defined? (ii) Have potential problems been anticipated and addressed? (iii) Is the statistical/analytical design appropriate and clearly explained?

All issues have been resolved.

5. **Research Capability** Comment on (i) the research team's expertise and track record (incl. principal investigator / project team members / collaborators) and (ii) the existing facilities of the Institution where the research will be conducted.

The team seems to have the capacity to undertake this research.

6. **Budget** Is the request for research personnel, consumables, equipment and overall budget justified and reasonable? [For reference, 1 USD is equivalent to approximately 7.8 HKD]

Seems appropriate to me.

7. **Ethical and Safety Considerations** Is the proposed research ethically sound? Outline any safety or ethical issues that from the proposed research and comment on whether these have been adequately addressed in the proposal. Has ethical approval been sought?

No ethical issues.

8. **Overall Comments and Conclusion** It is always helpful for applicants to receive constructive feedback from reviewers. What are the specific strengths and weaknesses of this proposal? Please include a brief overall appraisal of the proposal focusing on any areas for improvement and the basis for your comments, e.g. awareness of other work in the field.

Strengths:

This is an interesting, relatively simple, and inexpensive study that can help us understand the role of certain technologies in behavior modification.

Weaknesses:

I don't find any notable weaknesses.

REFEREE'S ASSESSMENT FORM

Reference No.: 23244181

Project Title: Effectiveness of Immersive and Embodied Enhancement via "Virtual Reality" on the Sustainability of Hand Hygiene Compliance: School-based RCT with 10-month Follow-up

PART A: REFEREE'S DETAILED REMARKS ON THE INDIVIDUAL SECTIONS OF THE GRANT APPLICATION

1. **Originality and Impact** What is the importance of the proposed research in terms of its originality and potential impact in the area under study? How will the research findings benefit patients and/or the healthcare system? Will the research findings improve patient care, population health, influence clinical practice and/or health services management, or inform health policy in Hong Kong and elsewhere? Have the potential facilitators and barriers to this impact being achieved been identified?

> As outlined by the applicants, VR has been shown to be an effective tool for behaviour change for other health issues. However, its use for promoting hand hygiene (HH) within school aged kids is limited. This study aims to evaluate the effectiveness of the tool on student HH compliance, as well as explore the factors impacting on the sustainability of the strategy. Given HH's important role in reducing transmission of pathogens and the fact that our behaviours are established as children, it makes sense to focus on this cohort. However one could argue that the focus should be on younger school children.

2. **Research Questions, Aims and Hypotheses** How specific, clearly expressed and realistic are the research questions, aims and hypotheses?

> Are appropriate for the proposed work.

3. **Subjects and Study Methodology** (i) Is the proposed design and methodology appropriate for the study? (ii) Are sample sizes clear, justified, adequate and realistic? (iii) Are any preliminary data available? (iv) How feasible is the proposed timeframe? (v) Please also provide comments on the following (where applicable):

- For proposals submitted under Advanced Medical Research (refer to Area of Project on page 1 of application), is this a clinical study which applies advanced technologies including but not limited to biotechnology in medicine, use of drugs and treatments, clinical trials, virtual health such as telemedicine, etc., to facilitate the translation of knowledge generated from health and health services or infectious diseases studies into clinical practice and to inform health policy?
- For proposals addressing thematic priorities under Implementation Science (refer to Section 5b of the application), are the appropriate framework(s) / model(s) with the pre-set criteria proposed to evaluate/assess the barriers and facilitators of implementation outcomes clearly stated?
- For Seed Grant proposals (i.e. grant ceiling is HK$500,000), is the prospect that a successful outcome will enable scale-up to a larger project/trial and/or enhance the efficacy/effectiveness of existing practice clearly stated and feasible?

> The update proposal takes into account the issues previously identified with the design. The trial will be a cluster design and the primary outcome will be measured via direct observation. The number of participants is 720 which will make it very challenging to collect all the observation points from, so the team need to consider this when designing the data collection tools and consider the processes used in clinical settings.

4. **Outcomes and Data Analysis** (i) Are the primary and secondary outcomes clearly defined? (ii) Have potential problems been anticipated and addressed? (iii) Is the statistical/analytical design appropriate and clearly explained?

> Are appropriate for what is proposed.

5. **Research Capability** Comment on (i) the research team's expertise and track record (incl. principal investigator / project team members / collaborators) and (ii) the existing facilities of the Institution where the research will be conducted.

> The team has the required expertise and track record for the proposed work.

6. **Budget** Is the request for research personnel, consumables, equipment and overall budget justified and reasonable? [For reference, 1 USD is equivalent to approximately 7.8 HKD]

> Is appropriate.

7. **Ethical and Safety Considerations** Is the proposed research ethically sound? Outline any safety or ethical issues that from the proposed research and comment on whether these have been adequately addressed in the proposal. Has ethical

approval been sought?

Nothing noted.

8. Overall Comments and Conclusion It is always helpful for applicants to receive constructive feedback from reviewers. What are the specific strengths and weaknesses of this proposal? Please include a brief overall appraisal of the proposal focusing on any areas for improvement and the basis for your comments, e.g. awareness of other work in the field.

Strengths:

The proposed work will fill a gap in our understanding of strategies that support HH behaviours.

Weaknesses:

I would have liked to have seen further evidence that the proposed intervention has been designed with the students.

REFEREE'S ASSESSMENT FORM

Reference No.: 23244181

Project Title: Effectiveness of Immersive and Embodied Enhancement via "Virtual Reality" on the Sustainability of Hand Hygiene Compliance: School-based RCT with 10-month Follow-up

PART A: REFEREE'S DETAILED REMARKS ON THE INDIVIDUAL SECTIONS OF THE GRANT APPLICATION

1. **Originality and Impact** What is the importance of the proposed research in terms of its originality and potential impact in the area under study? How will the research findings benefit patients and/or the healthcare system? Will the research findings improve patient care, population health, influence clinical practice and/or health services management, or inform health policy in Hong Kong and elsewhere? Have the potential facilitators and barriers to this impact being achieved been identified?

The proposal's originality is in using VR as a tool to contribute new basic science insight into the cognitive psychology of immersive embedded experiences. The application to hand washing and the school based sampling are means to that end. Children's nose and face touching behaviours make them re-acquire pathogen filled hands so rapidly after hand-washing that there is not much point in hoping for massive public health benefits for respiratory diseases from this research. Perhaps there are benefits from lowering spread of enteric disease by improving post-toilet hand-washing, but enteric disease control has not been a major public health priority in Hong Kong for decades.
Even if frequent and thorough hand-washing for school children was of massive public health benefit, the VR intervention is so capital and labor intensive, that it would not be a scalable tool for health promotion. However, maybe the basic contributions to health psychology could be applied to behaviour of far more public health importance like condom use, tobacco, or dietary choices.

2. **Research Questions, Aims and Hypotheses** How specific, clearly expressed and realistic are the research questions, aims and hypotheses?

Improved study design will appropriately design the cognitive content of the facts relayed in the immersive and non-immersive treatments to be exactly the same. It would help if a post-test could verify that knowledge quiz performance about the hand-washing facts could be used to show that both arms had the same average quiz score about the facts of hand-washing conveyed in the treatments. What if the immersive treatment created a higher or lower knowledge score due to changes in children's attentiveness? Unless one measures knowledge acquired it is not appropriate to attribute treatment effects to the immersion effect just because the knowledge delivered was comparable.
With the 0,3,6, and 12 month behaviour measures and a booster dose at 6 months the study has the opportunity to hypothesize different sizes of booster effects by comparing the differences between 12 and 6 month behavioural prevalence. It can also hypothesize differences in behavioural decay rates between 0-3 and 3-6 months. The 3 rd month check is nice to have, but if the budget is tight, it can be eliminated. Statistical analysis should be prepared to look for differential loss to follow up rates between the study arms and adjust for that based on statistical weighting.

3. **Subjects and Study Methodology** (i) Is the proposed design and methodology appropriate for the study? (ii) Are sample sizes clear, justified, adequate and realistic? (iii) Are any preliminary data available? (iv) How feasible is the proposed timeframe? (v) Please also provide comments on the following (where applicable):

- For proposals submitted under Advanced Medical Research (refer to Area of Project on page 1 of application), is this a clinical study which applies advanced technologies including but not limited to biotechnology in medicine, use of drugs and treatments, clinical trials, virtual health such as telemedicine, etc., to facilitate the translation of knowledge generated from health and health services or infectious diseases studies into clinical practice and to inform health policy?
- For proposals addressing thematic priorities under Implementation Science (refer to Section 5b of the application), are the appropriate framework(s) / model(s) with the pre-set criteria proposed to evaluate/assess the barriers and facilitators of implementation outcomes clearly stated?
- For Seed Grant proposals (i.e. grant ceiling is HK$500,000), is the prospect that a successful outcome will enable scale-up to a larger project/trial and/or enhance the efficacy/effectiveness of existing practice clearly stated and feasible?

It is a strength to propose 12 schools and 5 districts to sample from. The analysis should use this strength by testing for school and district level effect modifiers of the hand-washing outcome as well as locational effects on children's perception of peer endorsement of hand-washing. At present the peer effects are being collected as a qualitative interview. Peer effects are important impact multipliers and this revision of the proposal is improved by attending to them at least qualitatively.
There is still scope to improve the analysis by systematically collecting quantitative data on perceived peer compliance with and approval of hand washing.

4. **Outcomes and Data Analysis** (i) Are the primary and secondary outcomes clearly defined? (ii) Have potential problems been anticipated and addressed? (iii) Is the statistical/analytical design appropriate and clearly explained?

Self-reported handwashing has known limitations and investigators are aware. This version's use of direct observation and "Glo-Germ" is excellent. Actually measuring hand-washing knowledge can help to ensure that any differences between

study arms is due to immersion and not knowledge. The dynamic changes can be useful to track as outcomes.

5. **Research Capability** Comment on (i) the research team's expertise and track record (incl. principal investigator / project team members / collaborators) and (ii) the existing facilities of the Institution where the research will be conducted.

No concerns.

6. **Budget** Is the request for research personnel, consumables, equipment and overall budget justified and reasonable? [For reference, 1 USD is equivalent to approximately 7.8 HKD]

Budget is adequate.

7. **Ethical and Safety Considerations** Is the proposed research ethically sound? Outline any safety or ethical issues that from the proposed research and comment on whether these have been adequately addressed in the proposal. Has ethical approval been sought?

No concerns.

8. **Overall Comments and Conclusion** It is always helpful for applicants to receive constructive feedback from reviewers. What are the specific strengths and weaknesses of this proposal? Please include a brief overall appraisal of the proposal focusing on any areas for improvement and the basis for your comments, e.g. awareness of other work in the field.

Strengths:

Strengths are the presence of preliminary results and a track record in studying VR. The study is now tuned to study the basic question of how much immersive experiences motivate behavior change. Another strength is the ability to follow dynamics of effects' depreciation and amenability to boosting over time.

Weaknesses:

Weakness is that the study does not systematically measure perceptions of social norms and peer effects which can be powerful moderators of the immersive experience effects.

REFEREE'S ASSESSMENT FORM

Reference No.: 23244181

Project Title: Effectiveness of Immersive and Embodied Enhancement via "Virtual Reality" on the Sustainability of Hand Hygiene Compliance: School-based RCT with 10-month Follow-up

PART A: REFEREE'S DETAILED REMARKS ON THE INDIVIDUAL SECTIONS OF THE GRANT APPLICATION

1. **Originality and Impact** What is the importance of the proposed research in terms of its originality and potential impact in the area under study? How will the research findings benefit patients and/or the healthcare system? Will the research findings improve patient care, population health, influence clinical practice and/or health services management, or inform health policy in Hong Kong and elsewhere? Have the potential facilitators and barriers to this impact being achieved been identified?

This is a substantially revised proposal of an RCT to assess the impact of "Virtual Reality" intervention on the Sustainability of Hand Hygiene Compliance of school children. The use of VR for health promotion is relatively new, but builds some some previous research (e.g., the trial by Eichel et al. 2022). It has three potential impacts at a population level, but given the costs VR headsets will take time, costs, and logistics to deliver the intervention, particularly at scale.

2. **Research Questions, Aims and Hypotheses** How specific, clearly expressed and realistic are the research questions, aims and hypotheses?

The primary aim is to examine the effectiveness of the VR-based immersive hand-washing school programme for sustaining hand hygiene compliance and reducing infection-related absence and symptoms. The aims/ hypotheses could be clearer i.e. the study involves the collection of outcome data at four different timepoints (ranging from immediately after the intervention to 12 months post- intervention), including some after a VR treatment, video "booster" reminder (replay of last video experience).

3. **Subjects and Study Methodology** (i) Is the proposed design and methodology appropriate for the study? (ii) Are sample sizes clear, justified, adequate and realistic? (iii) Are any preliminary data available? (iv) How feasible is the proposed timeframe? (v) Please also provide comments on the following (where applicable):

- For proposals submitted under Advanced Medical Research (refer to Area of Project on page 1 of application), is this a clinical study which applies advanced technologies including but not limited to biotechnology in medicine, use of drugs and treatments, clinical trials, virtual health such as telemedicine, etc., to facilitate the translation of knowledge generated from health and health services or infectious diseases studies into clinical practice and to inform health policy?
- For proposals addressing thematic priorities under Implementation Science (refer to Section 5b of the application), are the appropriate framework(s) / model(s) with the pre-set criteria proposed to evaluate/assess the barriers and facilitators of implementation outcomes clearly stated?
- For Seed Grant proposals (i.e. grant ceiling is HK$500,000), is the prospect that a successful outcome will enable scale-up to a larger project/trial and/or enhance the efficacy/effectiveness of existing practice clearly stated and feasible?

As its primary outcome measure the study proposes using direct observation of hand-washing practice and computer-assisted method alongside surveys to measure self-reported hand hygiene compliance. The sample size calculation is based on 5-point discrete Likert scale when comparing treatment and control group in self-reported hand hygiene compliance, but it important to note that these outcomes are measured on four occasions, and so the question arises: which timepoints are used in the formal hypotheses testing?

4. **Outcomes and Data Analysis** (i) Are the primary and secondary outcomes clearly defined? (ii) Have potential problems been anticipated and addressed? (iii) Is the statistical/analytical design appropriate and clearly explained?

It is hard to understand why the sample size focus on self-reported compliance (A SECONDARY outcome) when direct observation is PRIMARY outcome. While the researchers have made efforts to address many of the reviewers' comments from the previous submission, it does not appear that the team have fully taken on the feedback in this aspect of the study design.

Given the potential cost of a VR intervention, it would be worth considering the feasibility of undertaking some type of economic evaluation alongside the RCT (or even an estimate of how much it would cost to scale the intervention up across HK). It is likely that such evidence would be required if the intervention was to be adopted into policy.

5. **Research Capability** Comment on (i) the research team's expertise and track record (incl. principal investigator / project team members / collaborators) and (ii) the existing facilities of the Institution where the research will be conducted.

The PA has undertaken previous work on VR, but the team appear to have limited RCT experience e.g. the PA list one trial list where they were part of a large team.

6. Budget Is the request for research personnel, consumables, equipment and overall budget justified and reasonable? [For reference, 1 USD is equivalent to approximately 7.8 HKD]

The budget is similar to the previous request, while the design has been simplified the move to cluster randomization comes at a cost in terms of power.

7. Ethical and Safety Considerations Is the proposed research ethically sound? Outline any safety or ethical issues that from the proposed research and comment on whether these have been adequately addressed in the proposal. Has ethical approval been sought?

None

8. Overall Comments and Conclusion It is always helpful for applicants to receive constructive feedback from reviewers. What are the specific strengths and weaknesses of this proposal? Please include a brief overall appraisal of the proposal focusing on any areas for improvement and the basis for your comments, e.g. awareness of other work in the field.

Strengths:

The team has worked hard to address the previous comments, and the study design appears to have been improved.

Weaknesses:

It is still unclear what is the primary outcome of the study (i.e. self-report or objective measure and at what time point) and how it has been used as the basis of the power calculations.
